# Bayesian Model Comparison and Parameter Inference in Systems Biology Using Nested Sampling

**DOI:** 10.1371/journal.pone.0088419

**Published:** 2014-02-11

**Authors:** Nick Pullen, Richard J. Morris

**Affiliations:** Computational and Systems Biology, John Innes Centre, Norwich, United Kingdom; University of Glasgow, United Kingdom

## Abstract

Inferring parameters for models of biological processes is a current challenge in systems biology, as is the related problem of comparing competing models that explain the data. In this work we apply Skilling's nested sampling to address both of these problems. Nested sampling is a Bayesian method for exploring parameter space that transforms a multi-dimensional integral to a 1D integration over likelihood space. This approach focusses on the computation of the marginal likelihood or evidence. The ratio of evidences of different models leads to the Bayes factor, which can be used for model comparison. We demonstrate how nested sampling can be used to reverse-engineer a system's behaviour whilst accounting for the uncertainty in the results. The effect of missing initial conditions of the variables as well as unknown parameters is investigated. We show how the evidence and the model ranking can change as a function of the available data. Furthermore, the addition of data from extra variables of the system can deliver more information for model comparison than increasing the data from one variable, thus providing a basis for experimental design.

## Introduction

Mathematical modelling has become an important tool in many areas of science and beyond as a means of summarising our current state of knowledge, challenging our understanding and making predictions. In the field of systems biology, mathematical models [1], [2] play a key role in finding patterns in ‘omics data, putting forward and evaluating hypotheses to help explain complex biological phenomena as well as guiding new experiments. Often the systems approach is a highly iterative process as models are generated, falsified, updated, validated, and refined as a function of increasing data. Numerous modelling approaches are used in practice, ranging from topological network structure analyses to stochastic partial differential equations on complex geometries. The techniques are appropriately aligned to the question at hand, the resolution one wishes to achieve, and the available data. In all but the simplest cases, a challenge to the modeller is the choice of a useful parameterisation of the problem and, often in discussion with experimentalists, to devise ways of obtaining reasonable estimates for the parameters of the system. Depending on the method, these parameters may be inherent to a machine learning approach, so-called black box parameters, and of little interest to the biologist or for mechanistic models they may actually correspond to biological entities such as concentrations, dissociation constants, or degradation rates that may be used for validation purposes and the design of further experiments. Recent approaches for performing parameter estimation include simulating annealing [3], spline techniques [4], regression [5], particle swarm [6], multiple shooting [7], and Bayesian approaches [8]–[10]. An effective method for parameter estimation is the Kalman filter technique, and recent variations of this method have been shown to perform well for examples of biological models [11], [12]. Overviews of some of these methods are available [13]–[16].

We focus here on dynamic mechanistic modelling for which the parameters themselves are of interest and not merely a means to an end. Many mechanistic modelling studies in biology have employed ordinary differential equations (ODEs) as the mathematical framework of choice. The reasons for this include the natural way that many biological problems can be posed as the study of the behaviour of a dynamic system of interacting components over time and the well-established numerical routines for solving such systems. For instance, converting a genetic regulatory network into a mathematical formalism can be achieved using established enzyme kinetics and following standard conventions [17]. This approach gives rise to a mechanistic model with (in principle) measurable, kinetic parameters. Unfortunately, however, these parameters are often unknown experimentally, or determined under *in vitro* conditions for analogous systems, and so have to be estimated from available data. This is a major hurdle that has received a lot of attention from systems biologists [14], [15], [18]. A common approach is to use optimisation algorithms [13] to find the best fit to the data [3], [14], [18]–[20]. This approach can be motivated by invoking maximum likelihood arguments. Local optimisation is very well established and numerous high-performance packages are available, often based around variants of Newton's method such as trust-region optimizers or conjugate-gradient approaches [21], nevertheless the non-linearity of biological systems can lead to multimodal fitness landscapes [22] that require global optimisation techniques to avoid getting trapped in local minima. Global optimisation [13], however, remains a challenge and despite a number of powerful approaches, such as genetic algorithms, simulated annealing and particle filters, finding a global optimum can rarely be guaranteed in practice. Furthermore, it has been noted that the global minimum may not result in biologically realistic parameters [23].

A known problem with maximum likelihood and, in general, optimisation approaches is that without further precautions they can lead to the overfitting of a model to the data, i.e. the parameters are far more sharply defined than is justified from the information content of the data [24]. These are well-documented problems with established solutions such as Bayesian methodology and information theory based corrective terms to maximum likelihood such as the Akaike information criterion (AIC) [25], [26]. A nice short review of these approaches applicable to systems biologists is given by Kirk *et al.* [27]. Another issue is that the best-fit set of parameters to a model may not be representative of parameter space [28]. An optimisation algorithm may miss important solutions or contributions from other parts of parameter space. Furthermore, it has been shown that in systems biology that not all parameters are uniquely identifiable [29]. There are issues of sloppiness and correlations between parameters [29], [30]. Parameters have also been shown to behave differently between corresponding deterministic and stochastic systems [31].

The scarcity of large quantities of high quality data is a common problem faced by computational biologists seeking to model an experimental system. The Bayesian framework [32], [33] is an attractive way of dealing with this issue in a way that reduces the risk of over-fitting. Bayesian inference naturally encompasses Occam's razor [34], [35] and so inherently accounts for the trade-off between the goodness of fit of a model and its simplicity [36]. The Bayesian approach doesn't aim to produce a point estimate for quantities of interest but captures the full uncertainty of the problem that is reflected in the posterior probability distribution. In particular for non-unimodal distributions point estimates can be misleading. Bayesian techniques are gaining interest in numerous research areas and finding increased application in computational biology [37], [38] due to the availability of state-of-the-art developments [8], [9], [22], [39]–[45]. Recent further advances have shown that multi-dimensional biophysical problems can be tackled successfully within the Bayesian framework; for example Markov chain Monte Carlo (MCMC) was employed for suitably approximating a prior distribution for studying the insulin secretion rate [46], thermodynamic integration for biochemical oscillations [22], and copula-based Monte Carlo sampling was used for comparing models of human zirconium processing [47]. However, the computational demands for such approaches often make them prohibitive for many problems. A main reason for this computational effort is in the calculation of high-dimensional integrals that arise through the process of marginalisation and normalisation in Bayesian inference [28], [32]. Monte Carlo techniques are the established way to compute such integrals, however, can require many thousands of cycles to deliver adequate results and there are known issues with MCMC sample decorrelation times [40]. Nested sampling [48] was put forward as a Bayesian variant of this approach and was shown to perform well for simple test examples [49]. Recently this approach has been used with success for astronomical data analysis [50], [51], for exploring configurational phase space of chemical systems [42], for parameter inference of a circadian clock model [52] and for one of the most challenging problems in biophysics, namely the exploration of protein folding landscapes [43].

In this contribution we explore the use of Skilling's nested sampling [48], [49] for biological models, an area that has received little exposure to this method to date [42], [43], [52]. Nested sampling has shown encouraging results and efficiency gains over other sampling techniques [50], [51], [53]. We show how the procedure produces samples from the posterior probability distribution of the parameters to compute the normalisation constant of the posterior, which is termed the *evidence* [28]. This evidence is used in the Bayes factor and hence in contrast to standard MCMC methods we obtain the key quantity for model comparison simultaneously with the posterior samples for parameter estimation. We demonstrate this approach with various biological models for sparse, noisy data.

## Methods

### Bayesian parameter inference

For parameter inference the task is to infer the probability over the parameters, 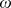, for the hypothesis or model, 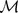, given some data 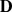 from an experiment and capturing also all relevant information 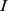. This can be done within the setting of Bayes' Theorem which states
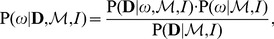
(1)where 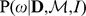 is the *posterior* probability, 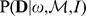 is the *likelihood*, 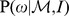 is the *prior* probability and 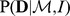 is the *evidence*. We make use of the following shortened notation [49]: 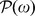 represents the posterior, 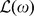 the likelihood, 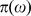 the prior and 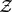 the evidence, hence (1) becomes
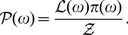
(2)Maximum entropy arguments lead to the assignment of a normal distribution for the errors in the data [33], and if the 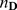 data points are independent the log-likelihood function resembles a least-squares residual
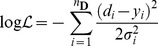
(3)where 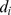 is the given data at timepoint 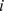, 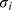 its corresponding standard deviation and 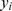 the value computed from the model at that point. More complex error models can be used if information is available or justified from the underlying experiment.

### Bayesian model comparison

Bayes' theorem not only enables us to infer parameter distributions but also provides a framework for model comparison. The posterior probability of a model 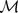 is
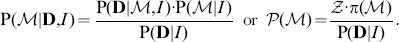
(4)To compare models we take the *posterior odds* of two models, 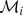 and 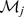, by taking the ratio and cancelling the term 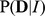. Thus
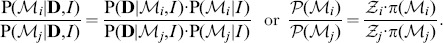
(5)If we have no prior preference for either model, i.e. 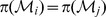, then these terms cancel out and the models are compared according to their respective evidences, which is identical to the normalisation constant in (1). This ratio of evidences is called the *Bayes factor* [54],
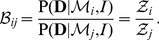
(6)Thus the evidence 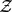 is the key quantity that can be computed by marginalising the likelihood 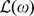 over parameter space,
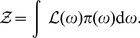
(7)


The evidence embodies the so-called Occam factor [28]. This is a measure of the extent to which the prior parameter space collapses to the posterior space after seeing the data. A model with more parameters typically has a greater volume of prior parameter space, and if the data are well described by only a small region of this space it will be penalised for this extra complexity. So a less complex model (fewer parameters) that fits well to the data for a larger region of its parameter space would be preferred by the Bayes factor calculation (Figure S1 in File S1). For most applications this quantity has to be estimated through the use of MCMC [55], [56], which is a computationally costly procedure for many Bayesian problems as high-dimensional numerical integration remains a challenge despite recent advances [53], [57].

### Nested sampling is a Monte Carlo technique constrained by the likelihood

Skilling [48], [49] showed that the evidence can be calculated by a change of variables that transforms the (possibly) multi-dimensional integral (7) over parameter space into a one-dimensional integral over likelihood space. Following Skilling [48], [49], we denote the elements of prior mass as 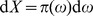 then 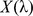 is the proportion of the prior with likelihood greater than 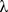 so that
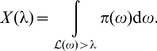
(8)The evidence can then be expressed as
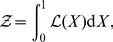
(9)where 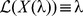. The basic algorithm proceeds as follows:

1. Sample the prior 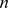 times to generate an active set of objects 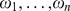 and calculate each object's likelihood.
2. Sort the objects based on likelihood.
3. Withdraw the point with lowest likelihood (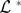) from the active set, leaving 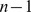 active samples.
4. Generate a new sample point from the prior subject to the likelihood constraint 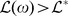.
5. Add the new sample 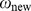 to the active set to return the set to 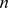 objects.
6. Repeat steps 2–5 until termination.

So by focussing on the evidence rather than the posterior distribution, a, potentially, high-dimensional integral can be replaced by a sorting problem of the likelihood [49], although high-dimensional sampling around each point remains. With the generated samples, the integral (9) can be approximated using basic quadrature as
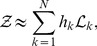
(10)where 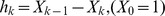 is the width between successive sample points and 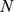 is the total number of samples i.e. the number of objects discarded from the active set plus those remaining in the active set at termination.

There is no rigorous termination criterion to suggest when we have accumulated the bulk of 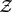 [49]. Skilling [48] suggests three ways and importantly notes that when to stop is a matter of user judgement. The MultiNest code [58] we make use of here terminates by approximating the remaining evidence that can be accumulated from the posterior. This amount can be estimated as 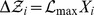, where 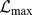 is the maximum likelihood value of the active set and 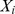 is the remaining prior volume [58], [59]. We use a tolerance of 0.5 in log-evidence as used in example problems [58] and found little difference in evidence estimates compared to using a higher precision of 0.1. In the materials applications of Burkoff *et al.* [43] and Partay *et al.* [42] they set their convergence criteria to reflect the nature of protein folding, based on the bounded nature of the energy, whereas Aitken & Akman [52] compare log-weight (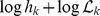) values 50 iterations apart.

**Figure 1:**
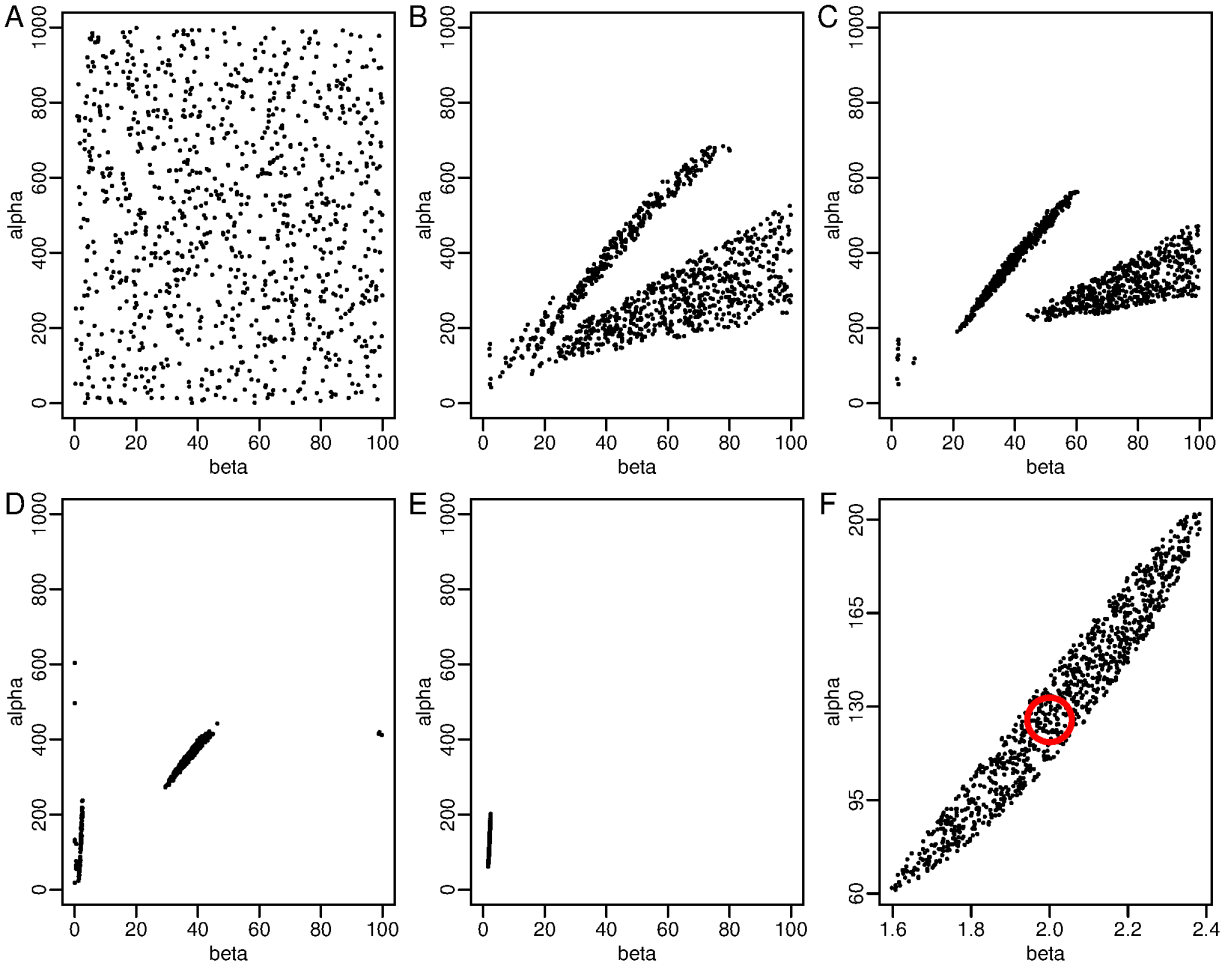
The migration of objects to higher likelihood regions. From an initial uniform parameter distribution (A), nested sampling selects points that are in regions of higher likelihood. The sample sets are shown after (B) 1800, (C) 2700, (D) 5400 and (E) 9000 sampling iterations, with (F) a close-up of the final (9000 iterations) sample set and the true value of the parameters indicated with a red circle (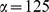 and 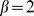). In this case, disconnected regions of high likelihood (B,C,D) are first explored before the sampling ends up in a single region of high probability (E,F). The underlying model is the repressilator, Equation (11), and the samples are from the posterior over the respressilator parameters 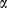 and 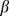.

With the constraint upon the likelihood, the method moves up the likelihood gradient to regions of higher likelihood even if these regions become disconnected in parameter space. This is demonstrated in Figure 1 (Figure S2 in File S1). As the algorithm moves through iterations there is a narrowing of the regions of higher probability as the worse samples are removed and better ones that satisfy the constraint on the likelihood survive. In this case all the objects left in the active set are located in one small cigar-shaped region of parameter space. This is where the bulk of probability mass is located for this model and data. This region includes the true parameters, Figure 1 F (red circle). If the procedure was run for even more samples, the objects would continue to move up towards the peak of the posterior probability distribution, and cluster closer together. The posterior parameter distribution allows for identification of possible areas where parameters are either stiff or sloppy. Figure 1 demonstrates how in one direction the posterior distribution is wide (sloppy) whereas in the perpendicular direction it is well defined (stiff). This example demonstrates the point made in Figure 1 of Erguler & Stumpf [29]. Disperse parameter sets are commonly found in systems biology yet can lead to useful predictions [30], [60]. Notwithstanding the technical difficulty of visualising multiple dimensions the multimodal posterior parameter space can reveal these regions of parameter space that lead to high probability yet may be disconnected. Using the posterior samples from nested sampling it is possible to gain an understanding of the underlying posterior distribution [49]. Staircase sampling can be used to generate equally-weighted posterior samples [49] (implemented by default in MultiNest [58]) and we make use of this later on.

For the nested sampling algorithm a greater sampling density from the prior distribution will increase the chances of highly probable areas being explored. In the study of protein folding [43] a set of 20000 prior objects was used to provide a wide selection of conformations. At the other end of the scale it has been shown that maintaining a set of 25 active points can produce accurate parameter mean and standard deviations that are relatively insensitive to the prior size [52]. All our results are based upon 1000 active points.

Estimating summary statistics of the posterior distribution is straightforward given the posterior samples from nested sampling [48], [49]. For example the mean 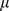 and standard deviation 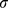 of a parameter 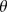 from 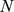 samples is calculated as
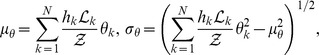
where each sample point, 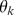, is assigned a weight, 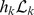, that corresponds to how much it contributed to the evidence. For our likelihood function choosing a larger value of 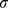 leads to greater evidence and larger variance of the inferred parameters in most cases.

### Implementation

All results in the following section used MultiNest (v3.0) [58] which can also perform the new importance nested sampling technique [59] (see also Table S1 in File S1). The Fortran wrapper around CVODE from the Sundials suite (v2.5.0) of ODE solvers [61] was employed as the main routine for solving the ODEs. All plots were produced using R [62] (> = v2.15.0) or ggplot2 [63] (v0.9.3.1). Pippi (v1.0) [64] was used for parsing the MultiNest output. The comparisons to MCMC were done using the PyMC library [65] (v2.3). Scripts are available from the authors.

## Results

### Nested sampling and MCMC

We compared the output of nested sampling with that of MCMC for Bayesian inference of two test problems. We compute the evidence and obtain posterior samples as a by-product within the nested sampling framework [48]. For MCMC, however, computing the evidence is known to be a complicated task [28], [48]. Other modern approaches that attempt to do this using MCMC are AIS and thermodynamic integration [22], [40], [53]. These approaches are reviewed by Friel & Wyse [66]. With nested sampling and MCMC we get the full posterior distribution and thus are able to quantify our uncertainty which we are unable to do with optimisation techniques.

**Figure 2:**
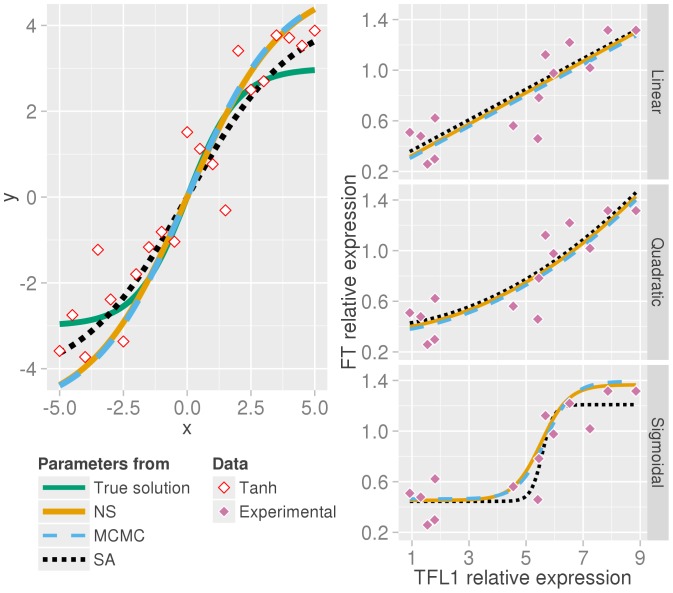
Nested sampling produces equivalent estimates to MCMC. (Left) Nested sampling (orange solid line) and MCMC (skyblue dashed) produce a similar estimate of the parameter means given noisy data (white diamonds) generated from 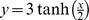 (green line). The solution using a point estimate of the parameters from simulated annealing is shown as a black dotted line. (Right) Using three different relationship models for flowering gene expression data nested sampling, MCMC and simulated annealing produce near identical estimates for a linear model of the experimental data (purple diamonds) and for a three parameter quadratic model. Curves are offset by one line width for clarity. For a four parameter sigmoidal model MCMC and nested sampling infer comparable parameter means (given in Table 1).

In the first case data were generated from the curve 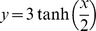 from 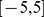 at intervals of 0.5 to give 21 data points. Noise from a standard Gaussian was added to the generated data. As expected from this low dimensional problem both nested sampling and MCMC find similar solutions with identifiable parameters whose means are good summaries of their distributions given the level of noise, Figure 2.

In the second example, we took expression data of the flowering time genes *TFL1* and *FT*, determined by quantitative PCR of the whole rosette in Arabidopsis upon the floral transition (Figure 2 [67]). Three different models between the antagonistic genes *TFL1* and *FT* are investigated: a linear model, a quadratic or a sigmoidal relationship. The measurement errors are not known but modelled as a normal distribution with 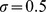 (data in arbitrary units) which we found to be consistent with estimated noise from the data (Figure S3 in File S1). We also used simulated annealing to optimise the parameters for a comparison with the means of our posterior parameter distributions. The fits to the data using the mean values for the three models are shown on the right in Figure 2. All methods find a very similar solution for the linear model, and equally for the three parameter quadratic curve. For the four parameter sigmoid model 
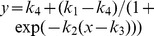
 the results are also comparable. The optimisation procedure fits the data well, with a steeper gradient than the inference methods. The means and standard deviations of the parameters from nested sampling and MCMC are in good agreement (Table 1). The log-evidences from nested sampling were found to be: 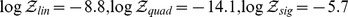, thus preferring the four parameter sigmoid model.

**Table 1:** Comparison of parameter means and standard deviations.

	Hyberbolic tangent		Linear		Quadratic			Sigmoid			
	*θ*_1_	*θ*_2_	*m*	*c*	*γ*	*β*	*α*	*k*_1_	*k*_2_	*k*_3_	*k*_4_
NS	5.05±1.84	0.26±0.24	0.12±0.05	0.20±0.26	0.01±0.02	0.02±0.23	0.37±0.44	1.37±0.53	2.06±1.42	5.53±2.00	0.45±0.22
MCMC	5.01±1.80	0.27±0.32	0.12±0.05	0.22±0.27	0.01±0.02	0.02±0.23	0.38±0.43	1.39±0.55	2.09±1.42	5.68±1.98	0.46±0.23
SA	4.36	0.24	0.12	0.22	0.01	0.02	0.38	1.21	5.00	5.57	0.44

The mean (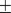 standard deviation) values of the parameters from nested sampling (NS), MCMC and the point estimates from simulated annealing (SA). The data came from 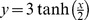 with additional noise and from Jaeger *et al.* (Figure 7 [67]) to which we fit three models: Linear 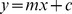; Quadratic 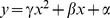; Sigmoid 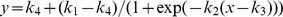.

### Nested sampling and parameter inference

The repressilator [68] is a frequently used system to evaluate parameter estimation developments [8], [11], [12], [69]. The repressilator is a synthetic network of transcriptional regulators comprising three genes in a feedback loop that is capable of producing oscillations. It is also the core structure of recent circadian clock models [70]. The governing equations used are as follows
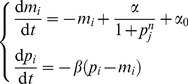
(11)where 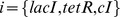 and 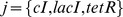. 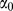 was set to 0 and 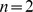 so that our prior contained both stable and unstable domains [68]. Initial conditions and parameters were chosen that produce oscillations in the synthetic data (Table 2). To show the power of nested sampling for this example we use data from just one variable, 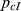 (cI protein), collected at two-minute intervals for 50 minutes. The data has Gaussian noise added to it with a standard deviation of 10% of the range. It is assumed we do not know, or cannot measure, the initial conditions for the five other variables, and attempt to infer these too. Uniform priors were used for all parameters with 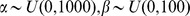 and the initial conditions are drawn from 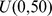. We choose a constant value of 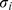 in (3) that is equivalent to the amount of noise added. When standard deviations can be estimated from the experimental data these values should be used in the error model. Either better quality (less noise) or greater quantity of data are both able to increase the accuracy of estimates of the parameter posterior probability distributions, as one would intuitively expect.

**Table 2:** Parameters and initial conditions of the repressilator model.

	*α*	*β*	*p_lacI_*	*p_tetR_*	*p_cI_*	*m_lacI_*	*m_tetR_*	*m_cI_*
True	125.00	2.00	5.00	0.00	15.00	0.00	0.00	0.00
Estimated mean	128.47	2.02	33.38	15.34	-	7.21	2.66	43.21
Estimated SD	5.88	0.05	8.46	10.73	-	5.26	1.73	4.67

The values of the parameters 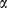, 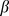 and initial conditions of the six variables used to generate the simulated data prior to addition of Gaussian noise, and the inferred means and standard deviations (SD) from the routine. 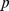: protein, 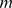: mRNA. The initial amount of cI protein was assumed to be known.

**Figure 3:**
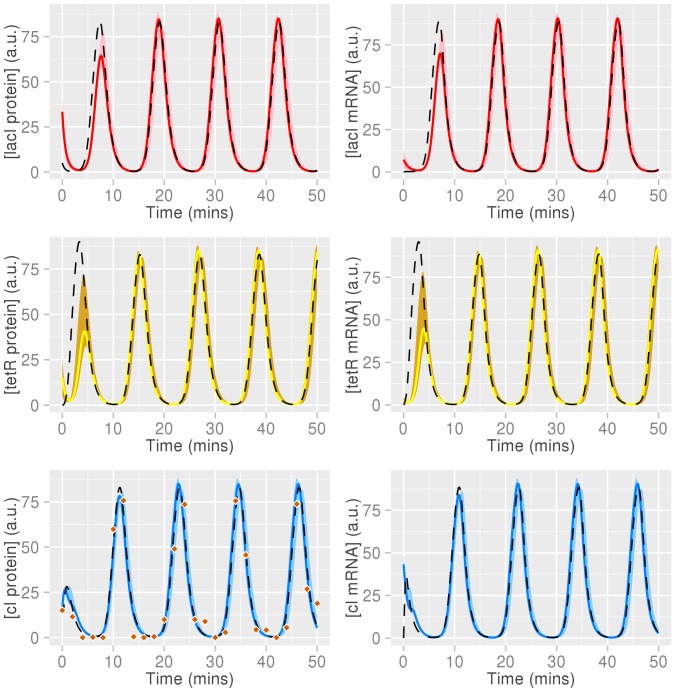
The inferred dynamics of the repressilator. Given just 26 noisy data points of cI protein (vermillion diamonds, bottom left) we were able to capture the full dynamics of the repressilator system with high accuracy, even when the mean estimates for the initial conditions of the other variables were not reflective of their true values. True solution, dashed black line; estimated dynamics using mean parameters, solid coloured lines; mean 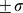, filled ribbons.

Using nested sampling we can produce an estimate of the means and standard deviations of the inferred parameters as explained in the Methods section. The actual values and inferred values are shown in Table 2. The system with these mean values as the actual parameters is shown in Figure 3 along with a ribbon representing 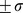. As can be seen, despite not estimating the initial conditions well, they are not that important for capturing the qualitative dynamics of the entire system. This is because the repressilator has a limit cycle and is therefore insensitive to most initial conditions. After the first peak the inferred oscillations match very closely to the true solution for all variables even though the algorithm only had a few, noisy data points available for one variable. The log-evidence for this model and data is 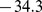.

A lack of accuracy in parameter estimations but well captured systems dynamics is a phenomenon that has been well studied in recent years [29], [30], [60]. In this case the unknown initial conditions and a lack of parameter identifiability has little overall effect on the quality of the reproduced data, whereas the two parameters 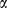 and 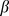 are estimated more accurately—the standard deviations in Table 2 are much lower relative to the prior size than for the initial conditions. Figures S4 & S5 in File S1 show the marginal and joint distributions for all parameters from this example. This enables us to see which parameters are more or less restricted and their correlations.

If we consider the model dynamics with 10 pairs of the parameters 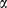 and 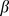 randomly drawn from the uniform prior there is a wide range of dynamics, Figure S6 in File S1, compared to the known solution (dashed black lines). In contrast, after the data have arrived, we can use the posterior samples to see how informative the data were about the parameters. Figure S7 in File S1 shows the dynamics from 100 posterior parameter sets. The data have constrained the parameter distribution significantly such that all sets closely match the true parameters' dynamics (dashed black lines).

In this example, the data significantly reduced the probable volume of parameter space from a wide prior distribution to a narrower posterior (Figure S8 in File S1). Even though the data were few and noisy the posterior distribution shows us that the data were informative enough to reconstruct the system's dynamics accurately.

### Nested sampling and model comparison

In this section we use synthetic data to compare four coupled ODE models:

- the Lotka-Volterra model of population dynamics [71], [72]

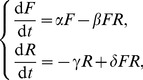
(12)
- the repressilator system in Equation (11),
- the Goodwin model of protein-mRNA interactions [1], [73]

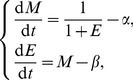
(13)
- the trimolecular two-species Schnakenberg model [2], [74]

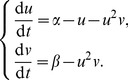
(14)

**Figure 4:**
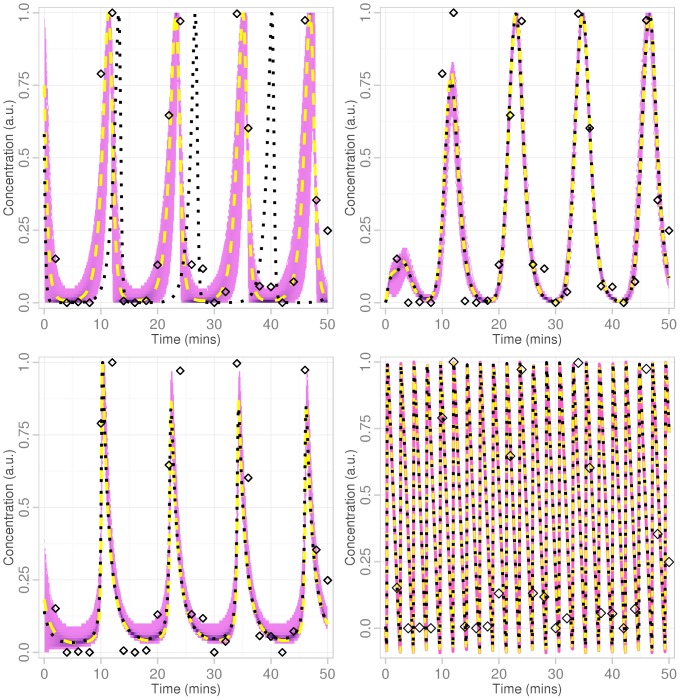
Fit to noisy data of four different oscillatory models. Clockwise from top left: Lotka-Volterra, repressilator, Goodwin and Schnakenberg models. Using the same noisy data (diamonds) 3000 equally-weighted samples (purple) were drawn from the posterior distribution of each model (except the Goodwin where we show a representative sample as all solutions were similar). The mean of the Lotka-Volterra system's posterior is not a good summary statistic for this distribution due to its non-unimodality (Figures S9 & S10 in File S1). The best-fit solution, dashed yellow line; solution using mean parameters, black dotted line.

We generate data from one variable of the repressilator system with known parameters, then add Gaussian noise. To ease comparison between different systems the data were scaled so that the amplitude is maximally one. All models are mechanistically different, however as all models are capable of oscillatory solutions, any of them could be used to describe the chosen data set if no further information was available. Our task is to evaluate if, and how well, we can choose between competing models given little data. Figure 4 shows 3000 samples from the posterior of all models (except Goodwin) along with the mean and best-fitting sample point from the four models. These fits for the Goodwin model have much higher frequency than the others, yet can still give a good least-squares error. Note that the concentration falls below 0 for this model with these parameters, which is clearly unbiological. The other three models pick out the correct frequency in the data. The mean of the Lotka-Volterra system, Figure 4, is not a good summary statistic for this distribution though the best-fit likelihood line for this model in Figure 4 shows a good fit to the data. This indicates care should be taken when summarising distributions. However merely relying on the best fitting parameters is essentially a maximum likelihood approach, and may miss important contributions from other parts of parameter space. To visualise this in Figures S9 & S10 in File S1 we show the marginal and joint distributions (with means and best-fit solution parameters indicated) for the Lotka-Volterra system which demonstrates the non-Gaussian shape of its posterior. The log-evidence values attained for the four models are shown in Table 3 indicating a very strong preference for the Lotka-Volterra model.

**Table 3:** Log-evidence of the four models for noisy data.

Model	log 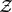
Lotka-Volterra	−23.4
Repressilator	−41.8
Schnakenberg	−44.8
Goodwin	−165.6

The log-evidence computed by nested sampling for each model using the 25 noisy data points shown as diamonds in Figure 4. Using the interpretation given by Kass and Raftery [54] the data provide very strong evidence for the Lotka-Volterra model (12) and against the Goodwin model (13) compared with the other models. The repressilator (11) has positive evidence for it over the Schnakenberg model (14).

**Figure 5:**
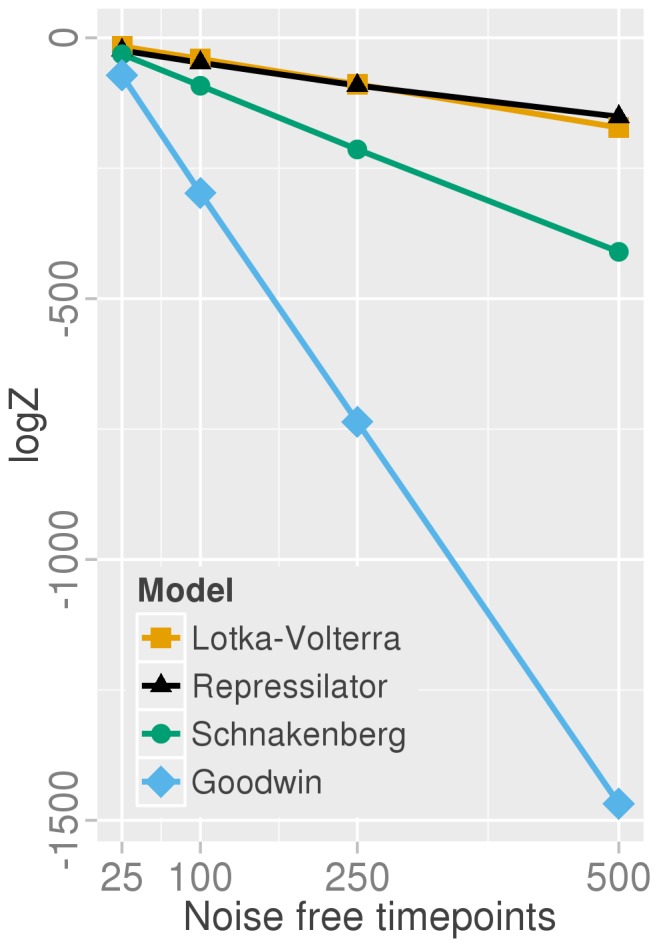
Evidence changes as a function of data quantity. As the resolution of the time course improves the Goodwin model (skyblue, diamonds) and the Schnakenberg model (green, circles) lose support faster than the Lotka-Volterra (orange, squares) and repressilator (black, triangles) systems. The known model—the repressilator—gains preference only for a larger number of data points (500 points with a time gap of 0.1), even when using noiseless data.

Given the nature of the sparse and noisy data it is not too surprising that a simpler model with two variables and six parameters is given preference over the model with six variables and eight parameters from which the data were actually generated. If the data are of better quality i.e. no noise and of greater density, we can see the repressilator model gaining more support (Figure 5) relative to the Lotka-Volterra system, but until an unreasonable amount of data is available (500 data points) the Lotka-Volterra model is preferred due to the it being the more parsimonious explanation of the data — visually both systems can fit the given data very well. Perhaps counter-intuitively, the evidence decreases with the increasing quantity of data. This is due to the log-likelihood function. As there are now more data points, unless the fit is exceptionally good, the least-squares residual increases due to summing up more errors. The evidence comprises both the Occam factor and the best fit likelihood (at least assuming the posterior is approximately Gaussian) [28]. Hence a worse likelihood score will similarly affect the evidence.

During this test we normalised the amplitudes and assumed none of the initial conditions were known, whereas in practice they can be normally be measured or taken to be the first time point. With the initial condition included for the repressilator variable measured, cI protein (as in Figure 3), and with unnormalised amplitudes, the log-evidence improved to 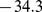 compared with 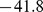 without knowledge of the initial point.

Taking fluorescence data from the original repressilator paper [68] as a proxy for one of the variables in the system it was investigated whether this was sufficient to support the known model. The data were extracted from Figure 2C [68] and a linear increase in fluorescence equal to 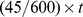 was removed. As the data are in arbitrary units it was rescaled to be maximally one again and the algorithm was used on the four models as before. Table 4 shows the results which now give positive to strong evidence for the Schnakenberg model. The experimental data and mean and best-fit parameter's solution are plotted in Figure S11 in File S1 which shows that although there is perhaps a fair fit in terms of residuals, in terms of the period of the data the posterior estimates are generally not at all close. If the frequency domain is known *a priori*, the likelihood function could be adjusted from a simple least-squares measure to take this into account.

**Table 4:** Log-evidence of the four models for experimental repressilator data.

Model	log 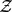
Schnakenberg	−101.7
Repressilator	−104.8
Lotka-Volterra	−124.2
Goodwin	−166.7

The log-evidence computed by nested sampling for each model using the 60 experimental data points given in Figure 2C of the original repressilator paper [68]. The linear increase in fluorescence with time was removed and as the original data is in arbitrary units, it was rescaled the data to be maximally one. Using the interpretation given by Kass and Raftery [54] the use of experimental data now provides positive to strong evidence for the Schnakenberg model against the repressilator and very strong evidence against the other two models.

If there was some uncertainty as to the model or its parameters, designing experiments that can maximise the information in the data is an approach that has been explored recently [75]. Experimentally it can be hard to increase the resolution of a timecourse so focussing on other genes or proteins of interest can be fruitful. With this in mind we looked at the effect of gathering data from another variable of interest rather than trying to increase the quantity of data available from one variable. As previously the repressilator system (11) was used to generate the timepoints, but now with two variables of 25 timepoints each and additional Gaussian noise. (The same random seed was used so as not to introduce this potential bias in generating the noise.) The four oscillatory models chosen before are used with nested sampling for model comparison. The results are presented in Table 5. There is now much stronger support, compared to just having data from one variable, for the repressilator model—the log-Bayes Factor has gone from 18 in favour of the Lotka-Volterra model over the other models to 72 in favour of the repressilator. This is regarded as decisive evidence for the repressilator [54]. For these example models the use of data from two variables gives far more information than increasing the quantity of data from one variable and enables us to prefer the known model. We are thus able to suggest this interesting aspect should also be considered when designing experimental research, and may be very useful for Bayesian model comparison.

**Table 5:** Log-evidence of the four models for noisy data from two variables.

Model	log 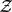
Repressilator	−77.4
Schnakenberg	−149.2
Lotka-Volterra	−339.1
Goodwin	−468.0

The log-evidence computed by nested sampling for each model using 25 noisy data points from two repressilator variables. For these example models, it was found that the use of data from two variables gives more valuable information than an increase in the quantity of data from one variable. Using the interpretation given by Kass and Raftery [54] the data provide decisively strong evidence for the repressilator.

## Discussion

Bayesian methodology offers a number of advantages over other inference techniques that include a consistent framework for including prior information and updating knowledge as more data become available, whilst appropriately accounting for the uncertainty in our inferences. The process of marginalisation is a powerful tool that enables parameters that are unknown or not of interest to be integrated out, thus allowing the Bayesian modeller to focus on key relevant quantities. The price to pay for these advantages is compute time. In particular for large problems that require the computation of complex integrals in high-dimensional space, this cost can be prohibitive.

Nested sampling is an effective way of calculating the evidence for a model and producing samples from the posterior distribution of the model's parameters. Nested sampling can be viewed as a Bayesian version of Monte Carlo for which initially the prior and then the likelihood are used to guide parameter space exploration. The 1D integral over the likelihood is solved by treating it as a sorting problem. As with other Bayesian approaches and in contrast to optimisation-based methods, samples are obtained from a full distribution of the parameters of interest rather than merely a point estimate for the parameter (and possibly an estimate of the variance depending on the method used). These posterior sample points can be used for further analysis. We have shown that the method of nested sampling can produce good estimates for the parameters in systems of ordinary differential equations under typical biological scenarios of sparse noisy data. Nested sampling was also shown to produce comparable parameter estimates to the established workhorse of Bayesian inference, namely MCMC, for a biological problem with experimental data. Nested sampling additionally has the advantage of calculating the evidence as its main focus, thus readily providing us with the quantity required for model comparison. For systems biologists this ability to achieve both parameter inference and model comparison with the same algorithm is clearly applicable to many current challenges in the field.

Using Bayes' theorem helps reduce overfitting. In our example above the plasticity of the Lotka-Volterra model meant that the single variable data set available was not good enough to prefer the repressilator model that the data were generated from. However when we introduced data from a further variable this was able to constrict the parameter space further to then convincingly prefer the repressilator. As the mechanisms of these two models are quite different the modeller may have background knowledge to prefer one system over another and certainly Bayes factors or any other metric for model comparison should not replace intelligent reasoning about the problem being studied.

Bayesian methods are growing in popularity amongst computational biologists and bioinformaticians [38] because they are suited to many varied problems; from short, noisy experimental time-series [10] to the problem of protein folding [43] as well as large data sets such as microarray data [39] or phylogenetics [37]. As more data, both in quantity and quality, becomes available to a Bayesian learning scheme this can be taken into account to update the posterior distribution over the parameters or model space. With the development of efficient routines, such as variants of nested sampling [59], [76] or thermodynamic integration [40] for calculating the evidence, Bayesian analysis is becoming more tractable and accessible.

If we have only a small number of models we wish to evaluate, the approach of separating each model to provide an individual prediction that can be used to guide experimental validation is tractable. Bayes factors can be used to compare and select amongst models. For prediction purposes, however, the full hypothesis space is of interest to take into account parameter and model uncertainty. Model averaging is thus an important concept that provides a canopy above the layers of parameter and model inference [28], [77]. In terms of the least biased prediction, multimodel inference is therefore the approach of choice [28], [37], [77], [78]. After the new data arrive, these can be used to update the probability distributions over each model's parameter space and furthermore to then update the probabilities of the models themselves by computing the posterior distribution over model space. Conceptually, we could be totally open about our choice of model space and consider an infinite number of models. In terms of prediction, the model itself could be seen as a nuisance parameter over which we need to integrate to marginalise and make inferences. This idea leads to a Bayesian neural network [79] and intriguingly the approach of integrating over infinite neural networks leads naturally to so-called Gaussian Processes [79], [80]. This methodology relies on the form of the covariance between data points but no longer on any specific network structure or single model of the underlying process, but is more akin to regression [28], [80]. Thus, if prediction rather than a specific model is the goal of the inference, the Bayesian framework would lead to approaches similar to those established in the machine-learning community. For biological systems of such complexity that we are unlikely to obtain sufficient data to robustly proceed with mechanistic modelling, black-box approaches may therefore be a fruitful and efficient way forward [16].

## Supporting Information

File S1 **Supplementary figures and tables.**
(PDF)
