# Supplementary material for: Bayesian Model Comparison and Parameter Inference in Systems Biology Using Nested Sampling
Source: PLoS One. 2014 Feb 11;9(2):e88419. doi: 10.1371/journal.pone.0088419 (PMC3921180; doi:10.1371/journal.pone.0088419)
Supplement: File S1 — Supplementary figures and tables. (PDF) [file pone.0088419.s001.pdf]

Bayesian Model Comparison and Parameter Inference in Systems Biology using Nested Sampling

Nick Pullen*, Richard J. Morris
Computational and Systems Biology, John Innes Centre, Norwich, United Kingdom
∗ E-mail: nick.pullen@jic.ac.uk

Supplementary Information

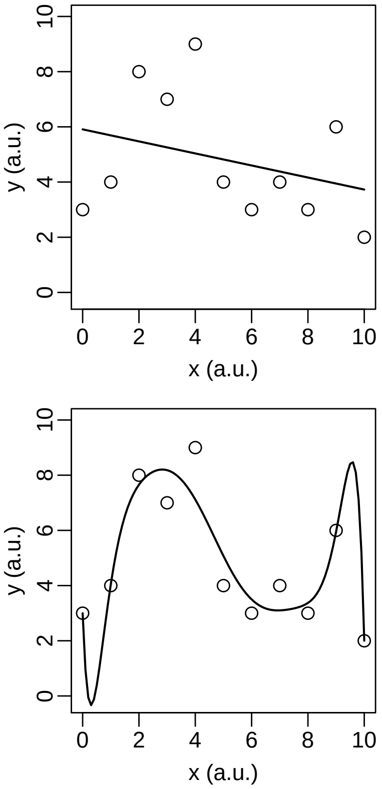

Figure S1. Optimisation can lead to overfitting. In terms of model comparison, optimisation alone may lead to an overly complex model being chosen as more flexibility often produces a better fit to the data with measures such as least-squares residuals. 11 data points, (x, y), are shown with (A) a linear fit that captures the overall trend, and (B) a 15th order polynomial fit that gives a fairly good fit to the data. The model fitting was carried out with a simulated annealing optimisation algorithm. The higher-order curve results in a much better least-squares residual of 7.4 compared to 48.4 for the linear model. The data points were generated from a uniform random number generator. Without further precautions, using the quality of the fit in terms of an optimisation residual for model comparison can be misleading. This is a well-known problem and numerous approaches have been develop to address this issue such as the AIC. The log-evidence we find for the complex model is -215.6 compared with -61.1 for the linear model. The log-Bayes Factor is thus over 150 in favour of the simpler model which can be interpreted as a very strong weight of evidence [1]. The fact that we need a complex model to achieve a good fit to the data is reflected in the probability of that model [2].

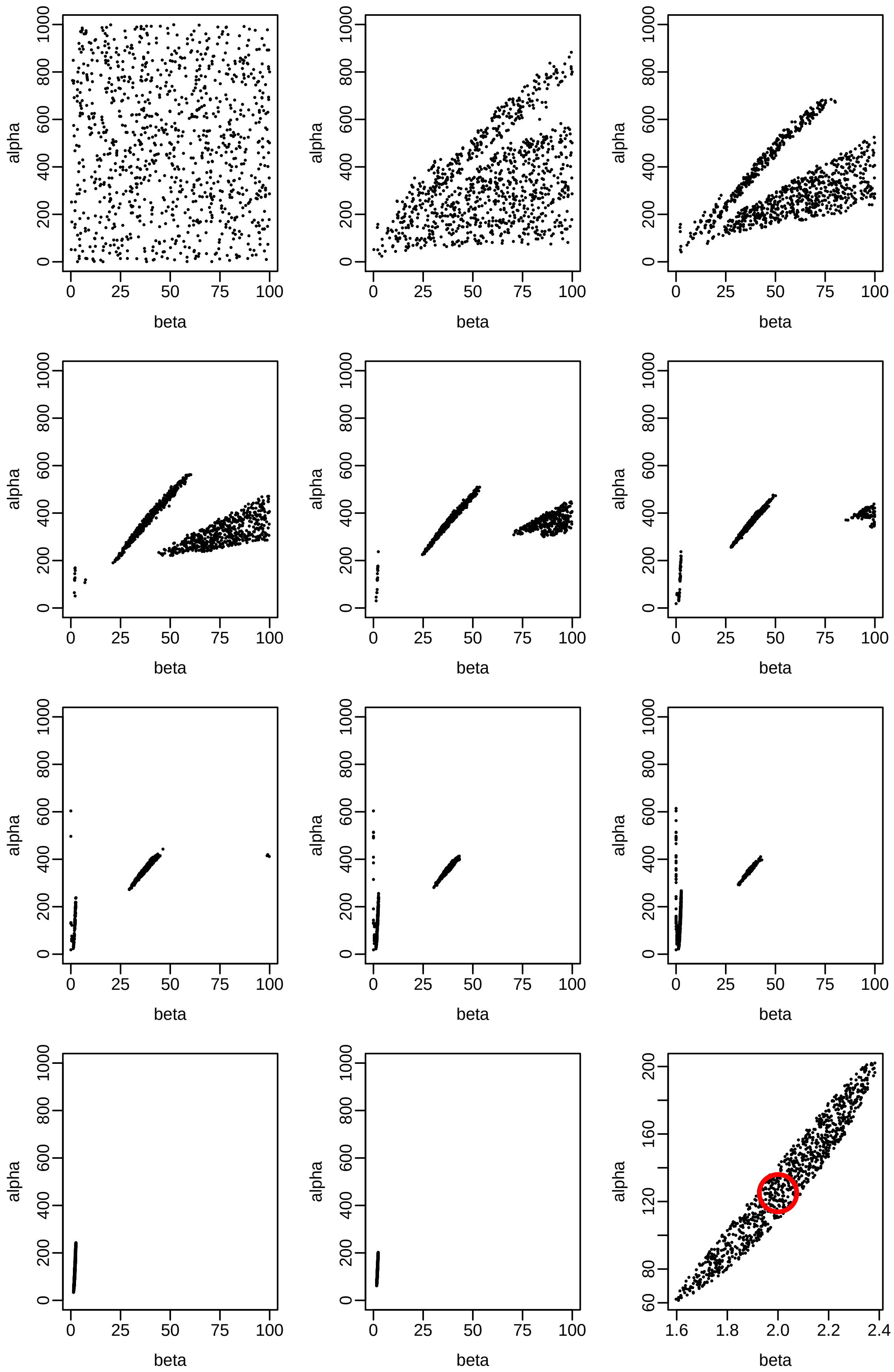

Figure S2. The migration of objects to higher likelihood regions. From an initial uniform parameter distribution, nested sampling [3, 4] selects points that are in regions of higher likelihood. The sample images are taken 900 sampling iterations apart, with a close-up of the final sample set and the true value of the parameters indicated with a red circle. Notice how despite disconnected regions of high likelihood, all sample points end up in the region of highest probability.

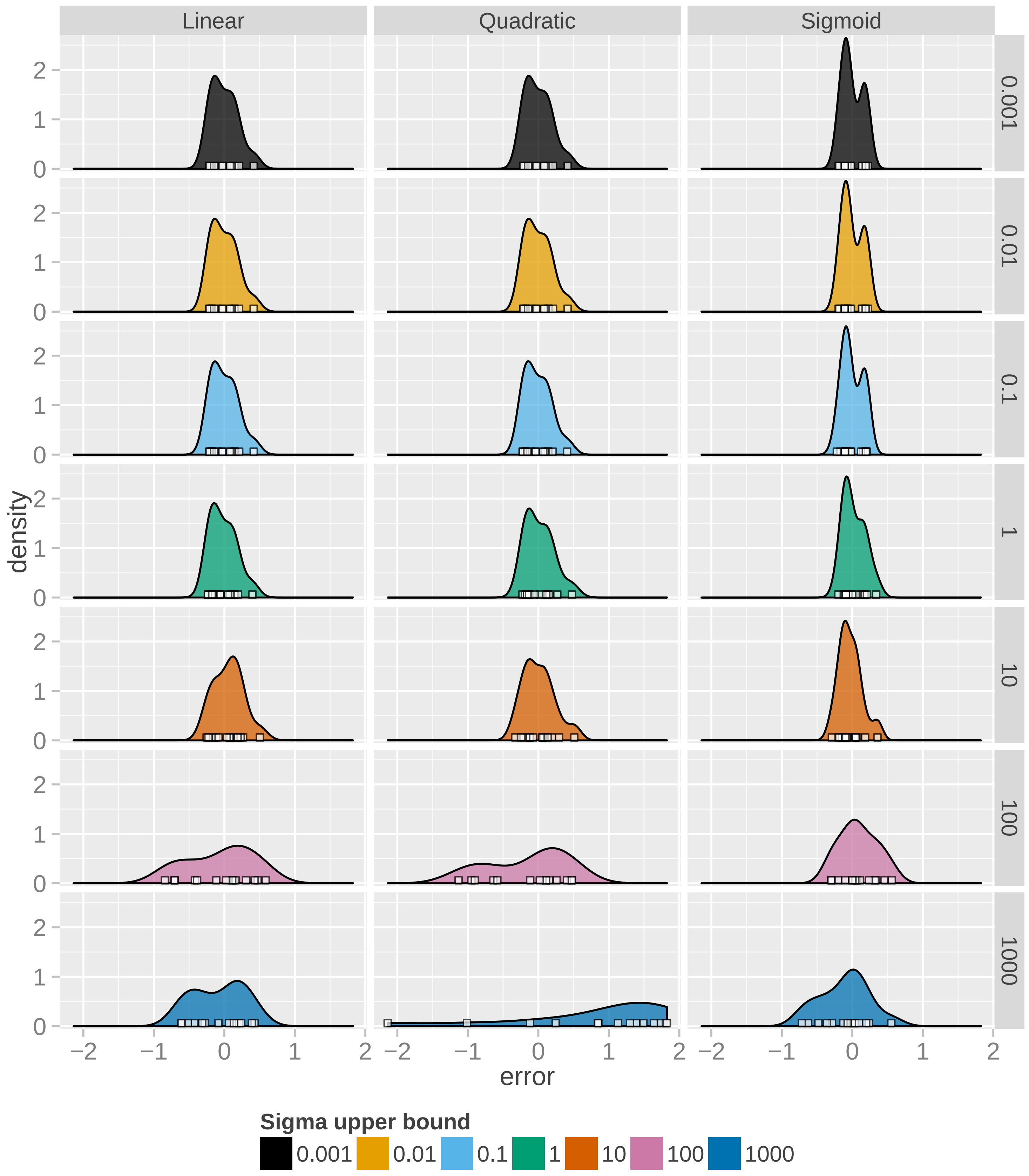

Figure S3. Kernel density estimates of the residuals by model and sigma upper bound. As an additional example we used nested sampling on the flowering time data [5] of the main paper but now sigma, rather than being set at a reasonable value, was inferred as a parameter that has a log-uniform prior in the likelihood function. The lower bound on sigma was 10^{-6} and the upper bounds are given in the figure. The sigmoid model, as the most complex with four parameters, shows the smallest errors as expected. Given the range of the data is a little over 1 the larger sigma vales are *a priori* highly unreasonable here and mean we are in effect capturing the whole of parameter space with our likelihood calculations. White squares represent the errors calculated as the difference between a data point (14 in total) and the best-fit solution.

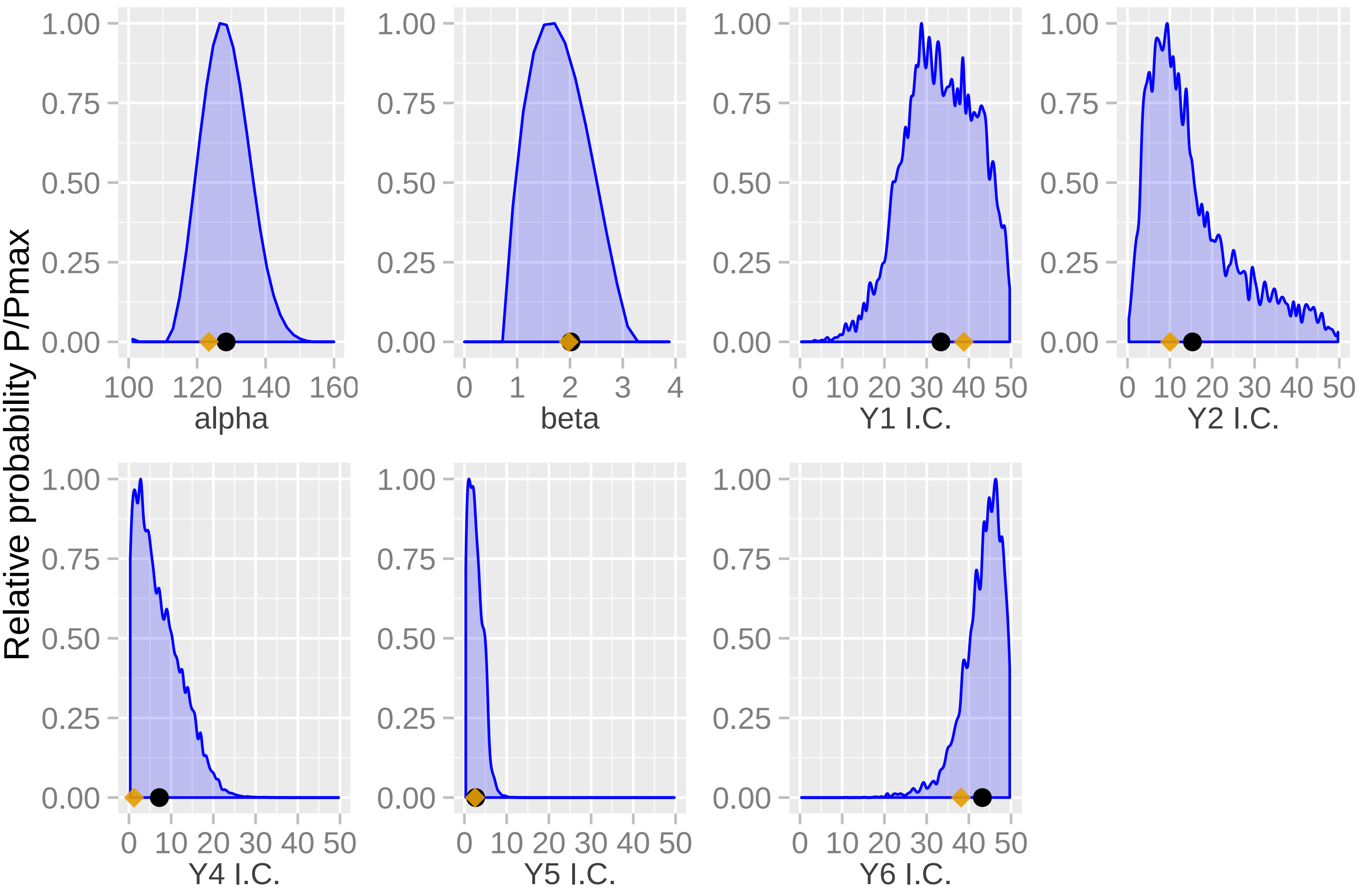

Figure S4. Marginal distributions of the repressilator example with missing initial conditions. Using the posterior samples produced as a by-product of nested sampling we can produce marginal distributions. In this example the mean and best-fit points are close to the peak of relative probability. Mean parameter value, black circle; best-fit likelihood parameter value, orange diamond.

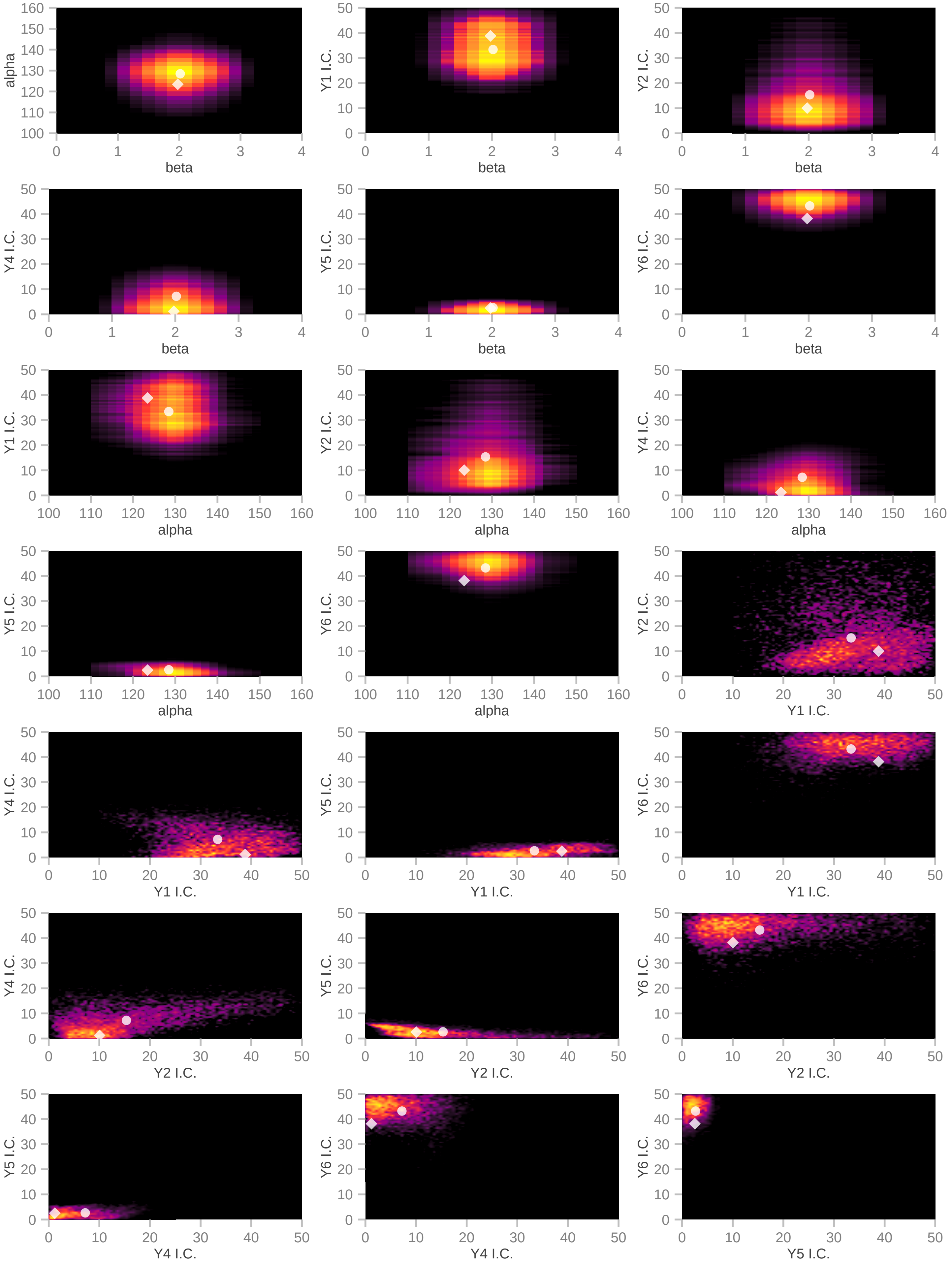

Figure S5. Joint distributions of the repressilator example with missing initial conditions. Using nested sampling we can also produce estimates of the joint distributions of pairs of parameters. Brighter colours indicate higher relative probability. Mean parameter value, white circle; best-fit likelihood parameter value, white diamond.

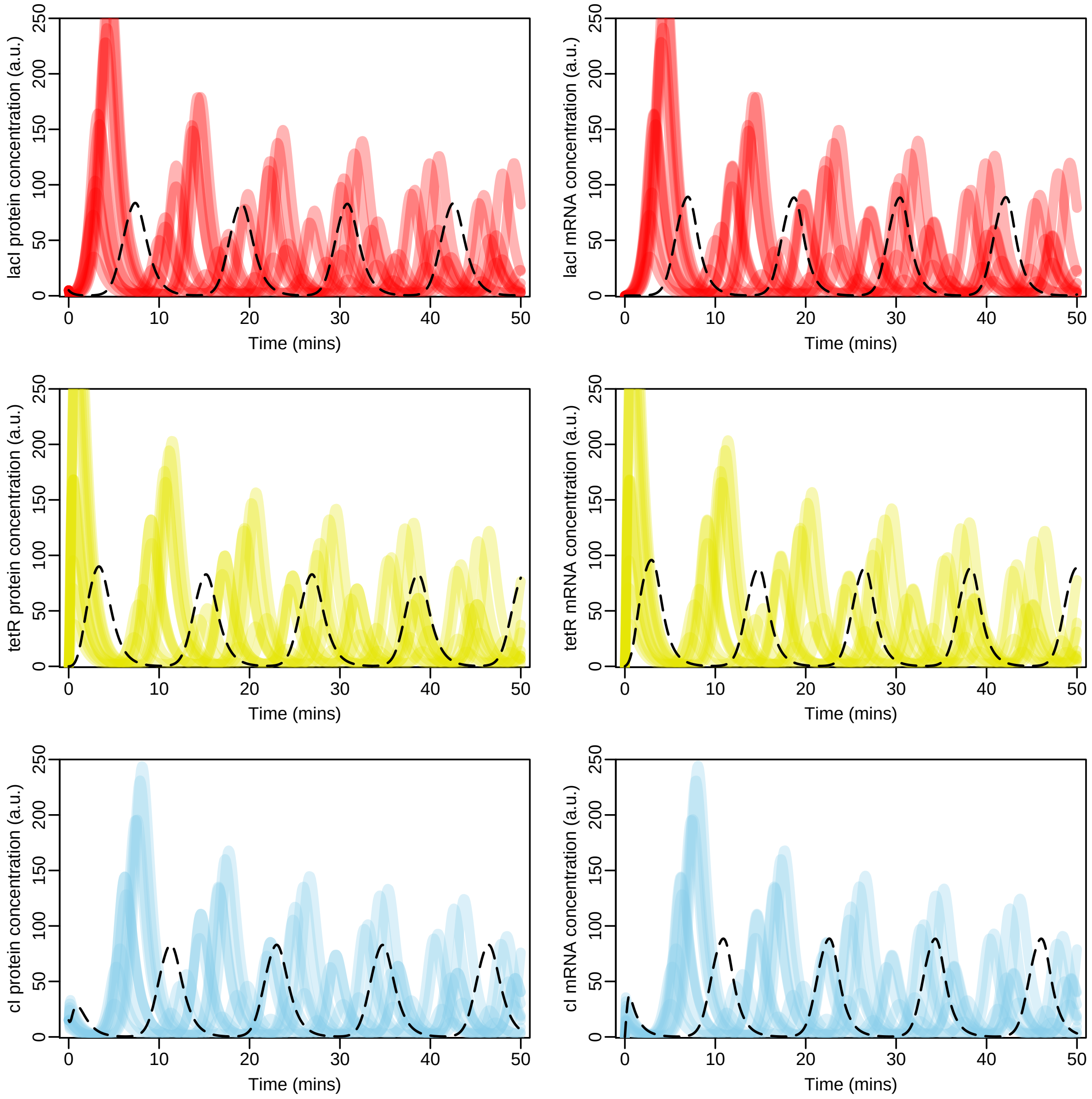

Figure S6. The dynamics of the repressilator with parameters sampled from the uniform prior. 10 different solutions of the system's six variables are shown with α and β chosen randomly from a uniform prior. Compared with Figure S7 the dynamics show a wide range of solutions. Solution with $\alpha = 125$ and $\beta = 2$ as in the main text, dashed black line; prior sampled dynamics, transparent coloured lines.

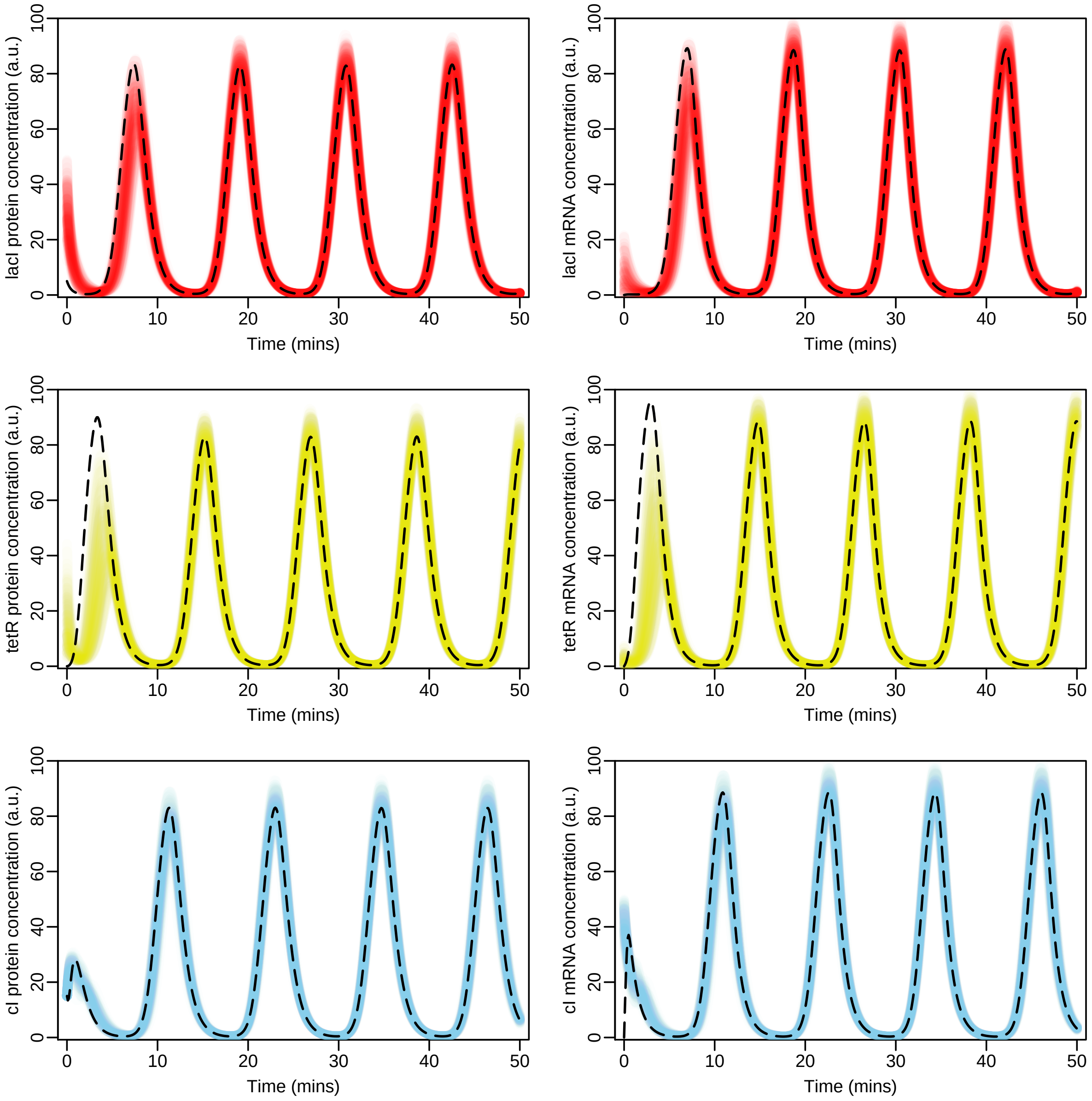

Figure S7. The dynamics of the repressilator with parameters sampled from the inferred posterior. 100 equally weighted posterior samples of the system's six variables are shown. Compared with Figure S6 the dynamics have been significantly constrained so that all solutions are close the the true solution (parameters given in the main text, dashed black line). Estimated dynamics, transparent coloured lines.

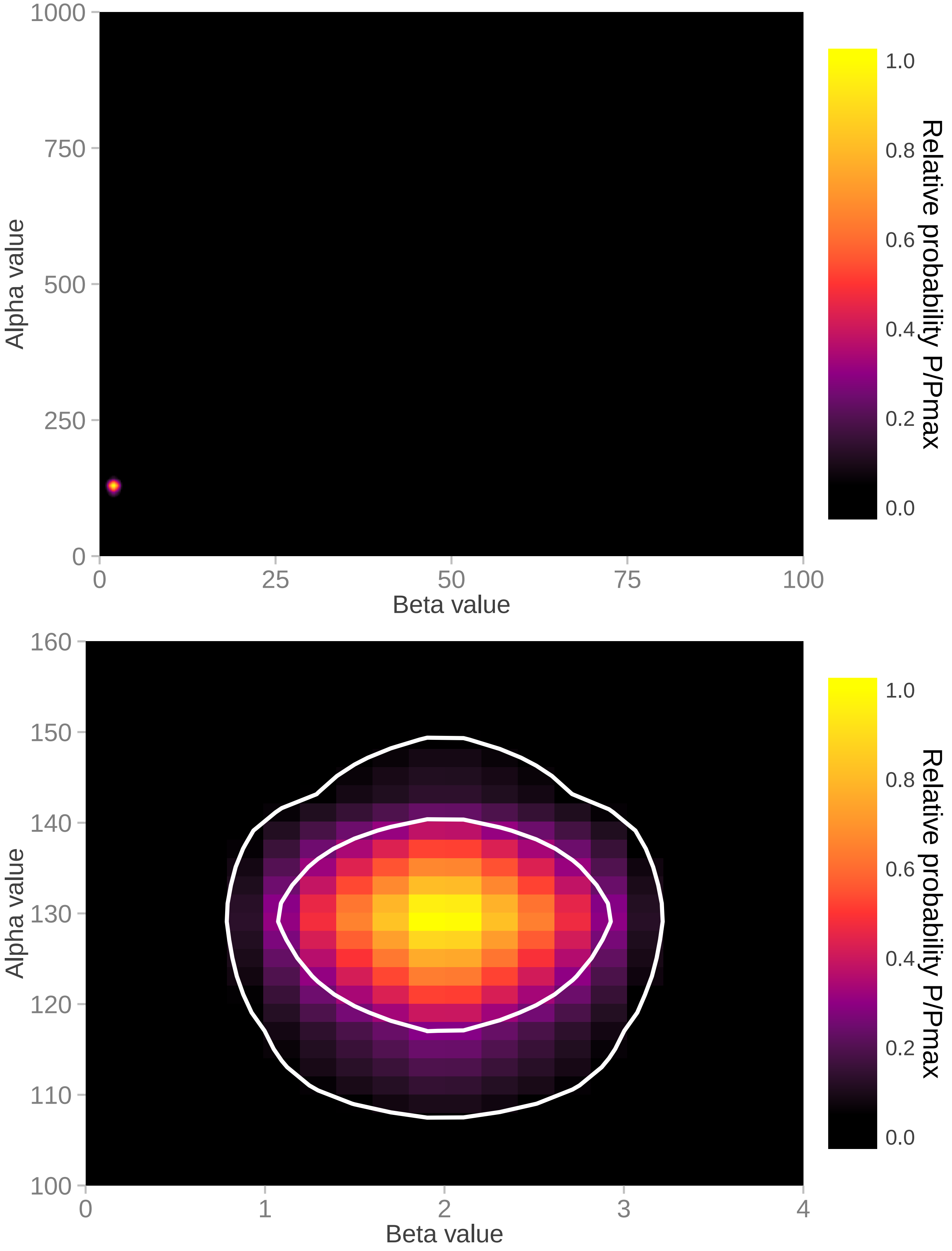

Figure S8. Nested sampling locates tiny regions of high probability. In the upper image we show the prior over α and β for our repressilator example with missing initial conditions. The posterior distribution is shown as a small speck in the lower left corner of the prior range. The lower image is a zoom in on the shape of the joint posterior which shows it to be unimodal. Nested sampling can locate this tiny region and provide accurate estimates for the parameters in doing so. Also shown are the contour levels at 68.3 and 95.4.

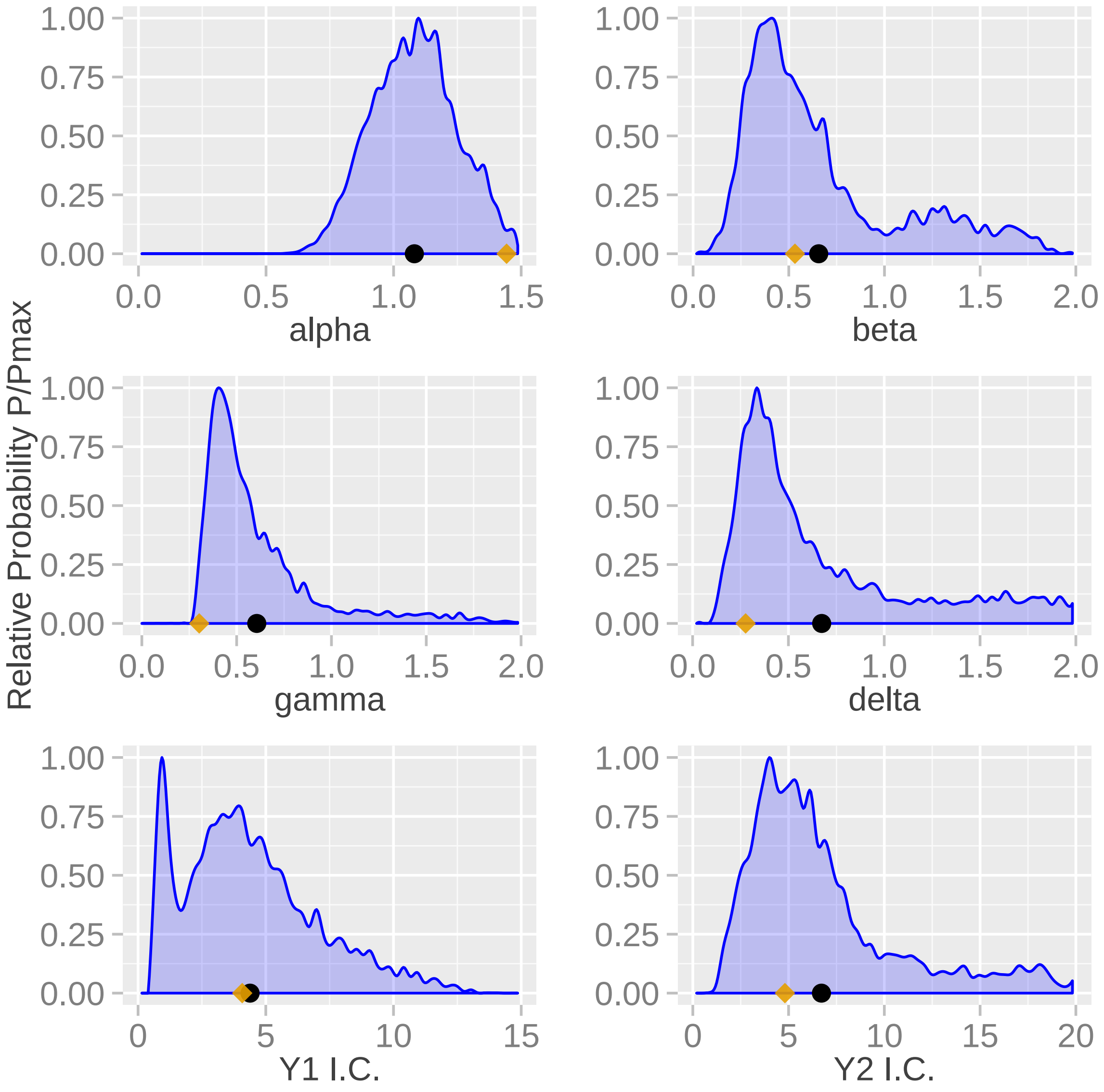

Figure S9. Marginal distributions of the Lotka-Volterra system. From the example of model comparison shown in the main text where 25 noisy data points generated from the repressilator system were used as the data for inference. Compared to Figure S4 parameters far away from the highest probability still have some probability and are accounted for in the Bayesian framework. Mean parameter value, black circle; best-fit likelihood parameter value, orange diamond.

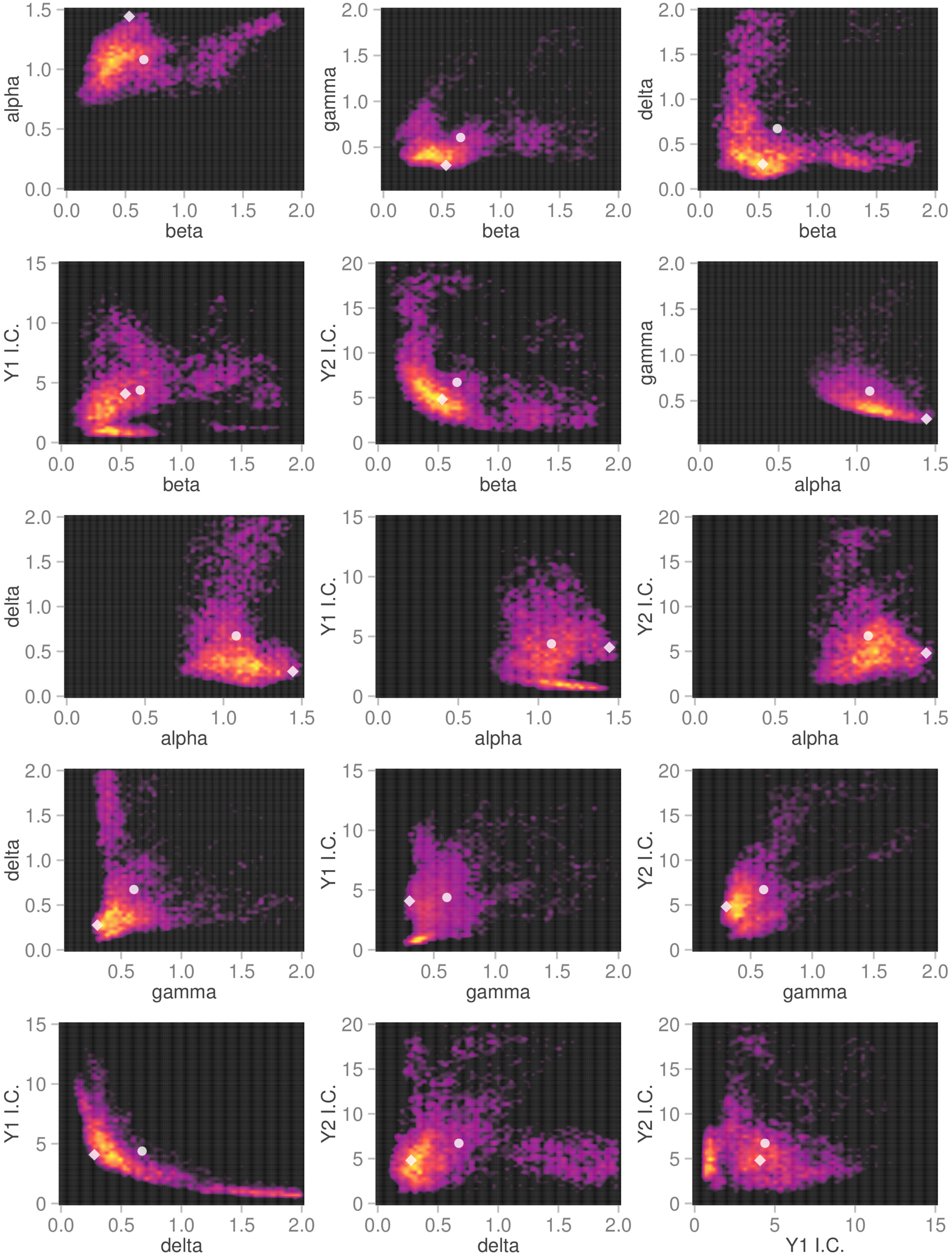

Figure S10. Joint distributions of the Lotka-Volterra system. From the example of model comparison shown in the main text where 25 noisy data points generated from the repressilator system were used as the data for inference. Compared to Figure S5 the joint probability landscapes are far less unimodal and provide an understanding of why the mean solution shown in the main article does not follow the same dynamics as the posterior samples. Brighter colours indicate higher relative probability. Mean parameter value, white circle; best-fit likelihood parameter value, white diamond.

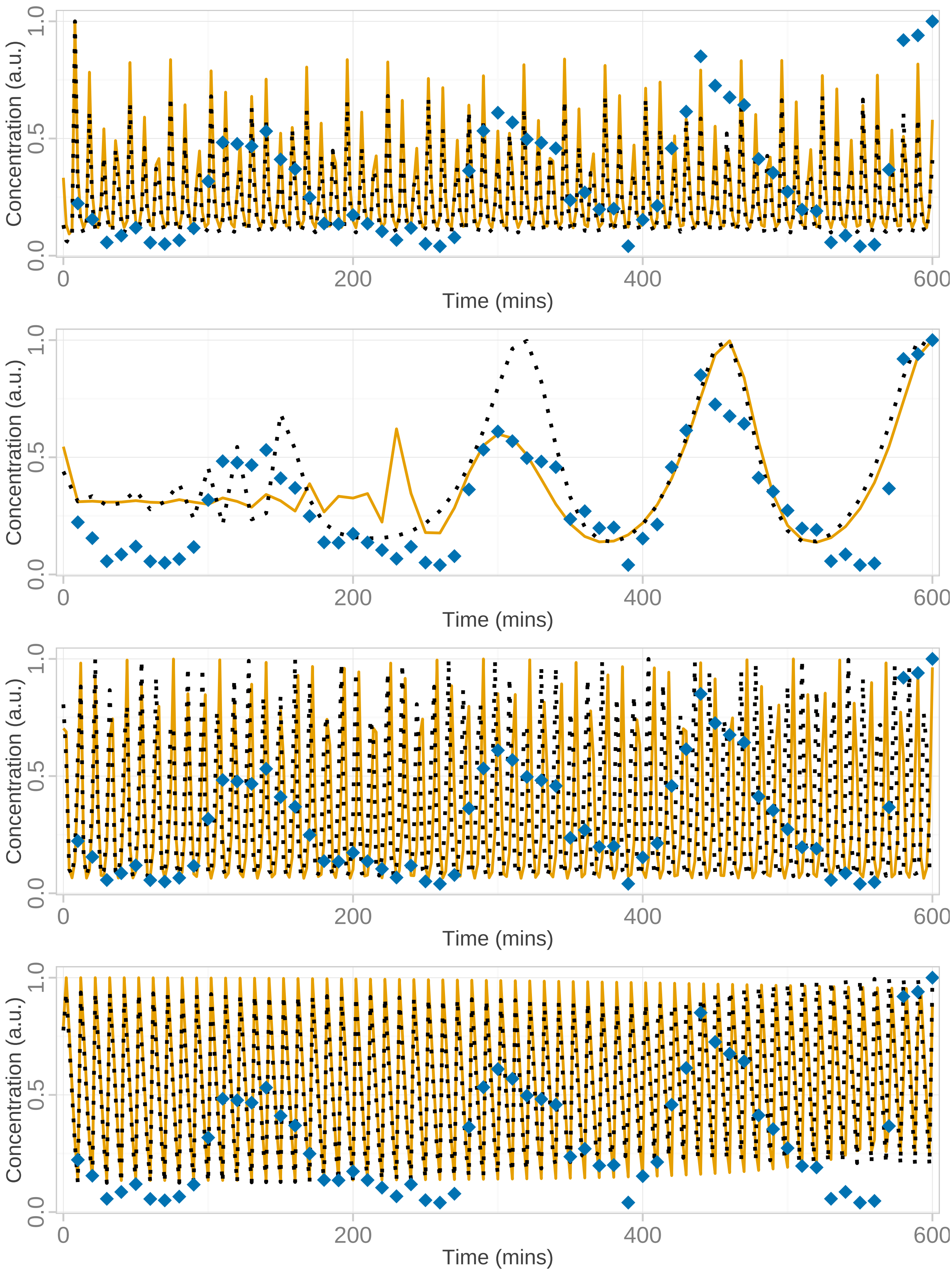

Figure S11. Mean and best-fit to the four models using experimental data. Experimental data points (blue diamonds) from the original repressilator paper [6] were used to compare the four models as in the main text. From top to bottom are the Schnakenberg, repressilator, Lotka-Volterra and Goodwin models. None of the models generally did well at identifying the correct period of the experimental oscillations. Thus when posterior samples were plotted it was hard to gain anything visually thus for simplicity we just show the mean and best-fit solutions. Towards the second half of the experimental time series the repressilator's mean and best-fit does match the data more closely but this wasn't the case for all posterior samples. Solution using mean parameter values, black dotted lines. Solution using best-fit likelihood parameter value, orange solid lines.

Example : Model	NS $\log \mathcal{Z} \pm error$	INS $\log \mathcal{Z} \pm error$
Hyberbolic tangent	-12.941 ± 0.069	-12.886 ± 0.258
Flowering time data: Linear Model	-8.846 ± 0.083	-8.868 ± 0.012
Flowering time data: Quadratic Model	-14.094 ± 0.109	-14.138 ± 0.017
Flowering time data: Sigmoidal Model	-5.738 ± 0.059	-5.683 ± 0.016
Repressilator without initial conditions	-34.275 ± 0.138	-33.950 ± 0.034
Noisy data from 1 variable: Lotka-Volterra	-23.410 ± 0.104	-24.841 ± 0.340
Noisy data from 1 variable: Repressilator	-41.816 ± 0.128	-42.229 ± 0.029
Noisy data from 1 variable: Schnakenberg	-44.843 ± 0.137	-45.257 ± 0.037
Noisy data from 1 variable: Goodwin	-165.596 ± 0.115	-166.151 ± 0.127
Noisy data from 2 variables: Lotka-Volterra	-339.074 ± 0.123	-340.884 ± 0.133
Noisy data from 2 variables: Repressilator	-77.437 ± 0.142	-77.232 ± 0.015
Noisy data from 2 variables: Schnakenberg	-149.170 ± 0.137	-149.177 ± 0.060
Noisy data from 2 variables: Goodwin	-468.026 ± 0.125	-468.830 ± 0.072
Noiseless 25 timepoints: Lotka-Volterra	-16.558 ± 0.105	-18.387 ± 0.122
Noiseless 25 timepoints: Repressilator	-24.192 ± 0.125	-24.451 ± 0.016
Noiseless 25 timepoints: Schnakenberg	-31.449 ± 0.135	-31.949 ± 0.027
Noiseless 25 timepoints: Goodwin	-71.780 ± 0.126	-71.759 ± 0.233
Noiseless 100 timepoints: Lotka-Volterra	-40.358 ± 0.130	-41.472 ± 0.025
Noiseless 100 timepoints: Repressilator	-47.824 ± 0.141	-47.858 ± 0.050
Noiseless 100 timepoints: Schnakenberg	-91.752 ± 0.142	-92.768 ± 0.010
Noiseless 100 timepoints: Goodwin	-297.302 ± 0.140	-297.698 ± 0.014
Noiseless 250 timepoints: Lotka-Volterra	-89.138 ± 0.134	-91.001 ± 0.043
Noiseless 250 timepoints: Repressilator	-91.539 ± 0.160	-91.406 ± 0.017
Noiseless 250 timepoints: Schnakenberg	-213.933 ± 0.146	-214.243 ± 0.016
Noiseless 250 timepoints: Goodwin	-736.161 ± 0.148	-734.868 ± 0.339
Noiseless 500 timepoints: Lotka-Volterra	-171.979 ± 0.144	-173.542 ± 0.030
Noiseless 500 timepoints: Repressilator	-151.346 ± 0.176	-151.521 ± 0.017
Noiseless 500 timepoints: Schnakenberg	-410.173 ± 0.152	-410.441 ± 0.014
Noiseless 500 timepoints: Goodwin	-1468.378 ± 0.160	-1467.259 ± 0.152
Experimental repressilator data: Lotka-Volterra	-124.187 ± 0.151	-123.977 ± 0.144
Experimental repressilator data: Repressilator	-104.847 ± 0.113	-107.980 ± 0.194
Experimental repressilator data: Schnakenberg	-101.663 ± 0.133	-102.544 ± 0.308
Experimental repressilator data: Goodwin	-166.696 ± 0.141	-166.960 ± 0.029

Table S1. All log-evidence results and numerical error. MultiNest [7] output for all examples in this paper using both Nested Sampling and Importance Nested Sampling [8]. Importance Nested Sampling uses the rejected points that don't satisfy the likelihood constraint for an alternative summation of $\log \mathcal{Z}$, see [8] for details of this recent idea. We give the associated numerical error with each result.

References

1. Kass R, Raftery A (1995) Bayes factors. Journal of the American Statistical Association : 773–795.

2. MacKay D (2003) Information Theory, Inference, and Learning Algorithms. Cambridge University Press.

3. Skilling J (2006) Nested Sampling for General Bayesian Computation. Bayesian Analysis 1: 833–860.

4. Sivia D, Skilling J (2006) Data Analysis: A Bayesian Tutorial. Oxford Science Publications. Oxford University Press.

5. Jaeger KE, Pullen N, Lamzin S, Morris RJ, Wigge PA (2013) Interlocking feedback loops govern the dynamic behavior of the floral transition in arabidopsis. The Plant Cell Online 25: 820-833.

6. Elowitz MB, Leibler S (2000) A synthetic oscillatory network of transcriptional regulators. Nature 403: 335–8.

7. Feroz F, Hobson M, Bridges M (2009) MULTINEST: an efficient and robust Bayesian inference tool for cosmology and particle physics. Monthly Notices of the Royal Astronomical Society 398: 1601–1614.

8. Feroz F, Hobson M, Cameron E, Pettitt A (2013) Importance nested sampling and the MULTINEST algorithm. arXiv preprint arXiv:13062144 .
